# Foster Care and Child Maltreatment Mortality Rates in the US

**DOI:** 10.1001/jamanetworkopen.2025.51677

**Published:** 2025-12-30

**Authors:** Frank Edwards, Kelley Fong, Robert Apel

**Affiliations:** 1 School of Criminal Justice, Rutgers University–Newark, Newark, New Jersey; 2 Department of Sociology, University of California, Irvine

## Abstract

**Importance:**

Approximately 2000 children die each year of child abuse and/or neglect. Foster care is designed as a targeted intervention to reduce harm to children, but its association with child maltreatment mortality is not well understood.

**Objective:**

To examine whether foster care entry rates are negatively associated with rates of child mortality due to child abuse and/or neglect.

**Design, Setting, and Participants:**

This cross-sectional study assessed associations between child maltreatment mortality rates and foster care entry rates at the state level using administrative data from all US states from January 1, 2010, to December 31, 2023 (N = 700 state-years). Administrative records on all children in state-supervised foster care and all children whose death was attributed by child welfare agencies to child maltreatment were assessed.

**Exposures:**

Number of children entering foster care per 1000 child population.

**Main Outcomes and Measures:**

Number of child fatalities due to child abuse and/or neglect per 100 000 child population.

**Results:**

The study analyzed 3.4 million records of children in state-supervised foster care from 2010 to 2023 and 24 108 child fatalities that states attributed to child abuse and/or neglect. In 2023, states reported a mean (SD) of 2.62 (1.85) deaths per 100 000 child population and reported a mean (SD) of 3.21 (1.75) entries into foster care per 1000 child population. No evidence was found of a negative association between state-year level foster care entry rates and state-year–level child maltreatment mortality rates (β = 0.17; 95% CI, 0.01-0.34).

**Conclusions and Relevance:**

In this cross-sectional study, child maltreatment mortality rates did not appear to decrease with higher foster care entry rates or increase with decreasing foster care entry rates. This evidence suggests that there is not a negative association between child maltreatment mortality rates and foster care entry rates.

## Key Points

**Question:**

Do state-level child mortality rates increase when foster care entry rates decrease?

**Findings:**

This cross-sectional study of 3.4 million records of children in state-supervised foster care found no evidence of a negative association between foster care entries and child maltreatment fatalities at the population level.

**Meaning:**

Current evidence suggests that decreasing rates of foster care entry are not associated with changes in child maltreatment mortality rates.

## Teaser

This cross-sectional study of children in state-supervised foster care assesses deaths due to child maltreatment and whether these fatalities are associated with foster care entry rates.

## Introduction

Approximately 2000 US children die due to child maltreatment each year, according to federal data, with a recent report calling these fatalities “the most tragic consequence” of child abuse and neglect.^1^ Prior research^1,2,3,4^ has focused on individual- and family-level characteristics associated with child maltreatment fatalities, such as child age, child sex, family and community poverty, child race and ethnicity, and household composition. However, less is known about the role of system-level factors, even as public officials tend to focus on the state child protection agency’s actions (or lack thereof) after child maltreatment fatalities.^5,6^ This study provides an analysis of the association between state-level trends in foster care entry and child maltreatment mortality using administrative data from all US states between 2010 and 2023.

States are authorized to remove children and place them in foster care when they believe caregivers’ actions or inactions are placing children in danger. Approximately 370 000 US children were in foster care on an average day in 2023.^7^ Foster care is envisioned as a last resort for children at imminent risk of severe harm due to child maltreatment^8^; such substantial government intervention into family life is justified by the presumption that the alternative, leaving children at home, would compromise their safety, potentially even leading to death in extreme cases.^9,10^ In 2018, the Family First Prevention Services Act opened up federal funding for services aimed at preventing children from entering foster care, creating new urgency for questions about how reducing foster care might affect child safety.^11^

Public commentary has suggested that recent reductions in foster care are a key contributor to increasing child maltreatment mortality rates.^12,13^ US foster care entry rates have decreased from 3.49 to 2.47 entries per 1000 children between 2018 and 2023, a relative decrease of approximately 29%.^14^ During the same period, reported child maltreatment fatalities increased from 2.43 deaths per 100 000 children in 2018 to 2.71 deaths per 100 000 children in 2023, an increase of approximately 10%.^15^

However, national trends mask substantial state-level variation. Each state operates a separate child welfare system, necessitating subnational analysis. To our knowledge, the association between states’ use of foster care and child maltreatment fatality rates has not been examined empirically. In this study, we analyze whether and how states’ foster care entry rates are associated with their child maltreatment mortality rates, examining evidence of a statistical association in a series of state-year level models using administrative data.

Rather than seeking to identify factors predicting individual child fatalities—the primary focus of prior research^2^—the current analysis takes a system-level perspective, examining the association of foster care with fatalities at the state level. Although we do not formally examine a causal relationship between foster care entry and child maltreatment mortality, our analysis aims to provide much-needed descriptive evidence regarding a critical public health concern—deaths due to child maltreatment—and the extent to which trends in these fatalities are associated with foster care entry rates.

## Methods

### Data Sources

The US Children’s Bureau provides estimates of all fatalities that states attribute to child abuse and/or neglect (24 108 fatalities documented between 2010 and 2023 for 694 state-years with complete data) in both annual child maltreatment reports^1^ and in the National Child Abuse and Neglect Data System (NCANDS) Agency File.^15^ For this cross-sectional study, we relied on NCANDS Agency Files for fatality counts for the years 2014 to 2023 and US Children’s Bureau Child Maltreatment reports for fatality counts between 2010 and 2013, prioritizing recent data revisions over initial data releases (for more details, see eAppendix 1 and eTable 1 in Supplement 1). Although NCANDS data are the most comprehensive national data on child maltreatment mortality currently available, they likely undercount maltreatment fatalities.^16,17,18^ We describe alternative data sources and the strategies we used to assess the impacts of underreporting on our conclusions in eAppendix 2 in Supplement 1.

We relied on the Adoption and Foster Care Analysis and Reporting System (AFCARS) for information on children entering foster care. AFCARS data are an annual census of all children in state-supervised foster care (3.4 million records for this period) collected by the states and reported to the US Children’s Bureau. We obtained child population counts from the National Cancer Institute’s Surveillance, Epidemiology, and End Results (SEER) Program, derived from the US Census Bureau estimates. We also relied on data from the American Community Survey 5-year samples archived as an annual time series by the National Historical Geographic Information System (NHGIS)^19^ for state-level demographic characteristics. This cross-sectional study was evaluated by and approved as exempt from review by the Rutgers University institutional review board (IRB); informed consent standards did not apply to this analysis of public and secondary administrative data as assessed by the IRB. We followed the Strengthening the Reporting of Observational Studies in Epidemiology (STROBE) reporting guideline.

### Measures

NCANDS classifies child maltreatment fatalities as a “death of a child as a result of abuse or neglect because either: (1) an injury resulting from the abuse or neglect was the cause of death; or (2) abuse and/or neglect were contributing factors to the cause of death.”^15^ All states report data on child fatalities linked to investigated child abuse and neglect cases. Many states report additional data on fatalities classified as maltreatment by child fatality review teams, medical examiners, or other professionals who were not linked to investigated child abuse and neglect cases.^1,18^ We discuss alternative data sources and the sensitivity of our results to variation in reporting standards in eAppendixes 2 and 3 in Supplement 1. Data on fatality counts are missing in 6 cases (0.8%). We joined counts of all documented maltreatment fatalities for each state and year with SEER-derived data on the size of the population younger than 18 years to compute our focal outcome variable: a state-year rate of child maltreatment fatalities per 100 000 children in the population.

The US Children’s Bureau provides detailed information on all children in foster care annually through the AFCARS foster care files. We used these data from January 1, 2010, through December 31, 2023, to count the number of children entering foster care at the state-year level. We joined these state-year counts of foster care entries to SEER population data to compute our focal measure, a state-year rate of foster care entries per 1000 children in the population.

Using NHGIS time series of the American Community Survey, we computed 3 additional population characteristics to adjust for potential confounding of variation in social disadvantage across places in the association between foster care and maltreatment fatalities. We obtained population counts of people 25 years and older who have not completed a ninth grade education, unemployed people in the labor force older than 16 years, and people living below the federal poverty level for each state and year between 2010 and 2023 and then divide by a state’s total population to compute a proportion of the full population for each of these measures.

We joined these variables to construct a 2010 to 2023 state-year panel (n = 700). For the 6 state-years that are missing data on child maltreatment fatalities (0.8% of total observations), we constructed 20 multiple imputation models to incorporate appropriate error into our estimates. There are no missing data on other measures.

### Statistical Analysis

We estimated a 2-way fixed-effects regression to evaluate state-level temporal associations between foster care entry rates and child maltreatment mortality rates. Our model can be expressed as follows:

*y_jt_* = α + β*x_it_* + θ*Z_it_* *+* *u_j_* + *u_t_* + *e_jt_*

where *j* indexes states and *t* indexes years, *y_jt_* is the maltreatment mortality rate per 100 000 population, *x_it_* is the foster care entry rate per 1000 population, and *Z_it_* is a matrix of state-year–level population characteristics. The error components include *u_j_*, representing fixed effects for states, and *u_t_*, representing fixed effects for years. The state fixed effects absorb time-stable differences in mean maltreatment mortality and foster care entries across states, and the year fixed effects absorb annual national fluctuations in maltreatment mortality and foster care entries. *e_jt_* is a state-year error term. The coefficient β measures variation in maltreatment mortality associated with increase or decrease in foster care entries (net of unobservable variables peculiar to states and years) and is our focal estimand. The standard errors are clustered by year and by state.

We expanded this basic model several ways to probe for sensitivity, including specifications adjusting for temporal autocorrelation, spatial dependencies, and nonlinear functional forms. As child maltreatment fatality data are often underreported,^16,18,20^ we developed a series of sensitivity analyses using simulated data under varying conditions of bias and measurement error in mortality rates.^21,22^ We describe these methods and their results in eTables 2, 3, and 4 and eFigure 2 in Supplement 1. We rely on a conventional *P* < .05 threshold for statistical significance. Statistical analysis was performed with Stata, version 19 (StataCorp) and R, version 4.5.1 (R Foundation for Statistical Computing). A replication package is available online.^23^

## Results

We analyzed 3.4 million records of children in state-supervised foster care from 2010 to 2023 and 24 108 child fatalities that states attributed to child abuse and/or neglect. Most children whose deaths are attributed to child maltreatment were very young. In 2023, for the subset of states that provide detailed information on fatality cases, 707 children (44%) who died were younger than 1 year at the time of their death and 1323 (82%) were younger than 6 years when they died.^1^ Foster care entry was also most common for very young children. In 2023, 33 572 children who entered foster care (19%) were younger than 1 year at the time of entry; 82 443 children entering foster care (47%) were younger than 6 years.^14^

At the national level, child maltreatment fatalities were at a period minimum of approximately 2.0 deaths per 100 000 children in 2013 and a maximum of approximately 2.8 deaths per 100 000 in 2022. Foster care entry rates were at a national maximum of 3.7 per 1000 children in 2016 and a minimum of 2.5 per 1000 children in 2023. However, these national trends mask subnational heterogeneity. The Figure displays child maltreatment mortality rates (per 100 000 children) and foster care entry rates (per 1000 children) for 2010 to 2023 at both the national and state levels. The top panel shows national-level mortality rates per 100 000 children alongside foster care entry rates per 1000 child population.

**Figure.**
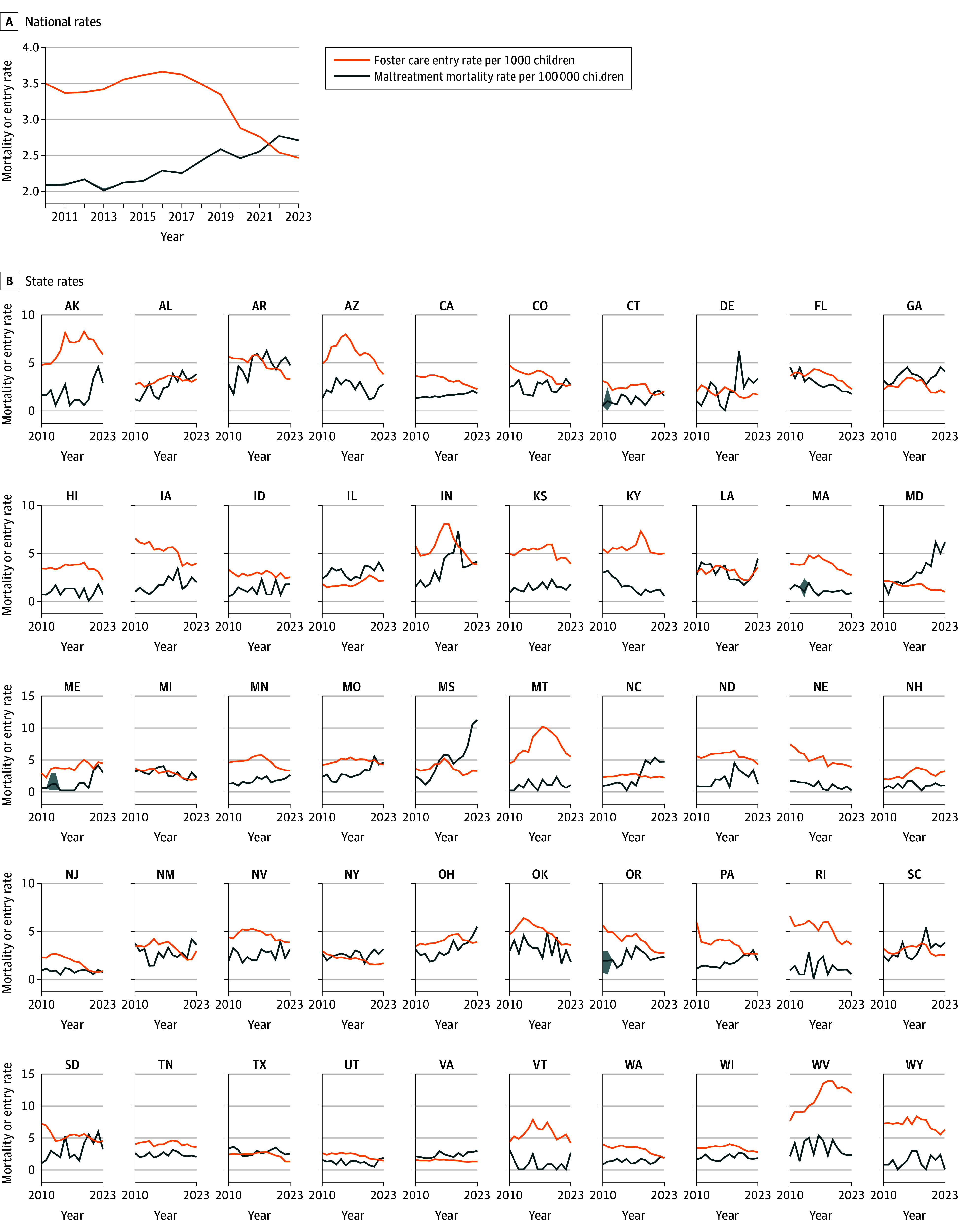
Reported Child Maltreatment Mortality Rates and Foster Care Entry Rates, 2010 to 2023 Foster care data are from the Adoption and Foster Care Analysis and Reporting System, and mortality data are from the National Child Abuse and Neglect Data System (NCANDS). Shaded area represents the postimputation 95% CIs.

The Figure also illustrates the variability of maltreatment mortality rates within and across states. Because maltreatment fatalities were a relatively rare event, large fluctuations in year-to-year mortality rates within states were common. Reported state child maltreatment mortality rates generally fell between 0.4 (fifth percentile) and 4.6 (95th percentile) deaths per 100 000 children per year between 2010 and 2022, with a median (IQR) mortality rate of 2.0 (1.2-2.9) deaths per 100 000. A small group of states reported mortality rates greater than 5 per 100 000 children during this period: Arkansas (2015-2022), Delaware (2019), Indiana (2019), Maryland (2021 and 2023), Missouri (2021), Mississippi (2016-2023), North Carolina (2021), South Carolina (2019), South Dakota (2015, 2020, and 2022), and West Virginia (2016). In 2023, Mississippi had the highest rate of reported maltreatment fatalities during this period: 11.2 deaths per 100 000 children. Mortality rates generally increased across states during this period: 13 states had mortality rates in 2023 that were less than or equal to reported 2010 mortality rates, whereas 37 had higher mortality rates in 2023 than in 2010. However, mortality rates were subject to substantial year-to-year variation and did not follow clear linear trends.

Nationally, foster care entry rates were at a period minimum in 2023 at 2.5 entries per 1000 children and at a period maximum of 3.7 per 1000 in 2016. However, as with maltreatment fatalities, national trends mask substantial state-level variation. During this period, 90% of state-level foster care entry rates were between 1.5 and 7.3 entries per 1000 child population. West Virginia consistently led the nation in foster care entry rates, with a period maximum of 13.8 in 2018 and rates above 10 per 1000 child population between 2015 and 2023. New Jersey reported the lowest rates of foster care entry in the nation, with a minimum of 0.7 entries per 1000 child population in 2022. Foster care rates generally decreased across states during this period: 10 states saw higher foster care entry rates in 2023 compared with 2010, whereas 40 saw lower rates in 2023 compared with 2010. Like national trends, state trends were not clearly linear. Many states saw both substantial increases and decreases in entry rates between 2010 and 2023.

To formally evaluate associations between foster care entry rates and reported child maltreatment mortality rates, we estimated a 2-way fixed effects regression with controls for variation in population disadvantage across places and times (Table). We found no evidence for a negative association between foster care entry and documented maltreatment fatalities. We estimated the association between foster care entry rates and maltreatment fatality rates at 0.17 (95% CI, 0.01-0.34) after adjusting for time-stable state features, national period effects, and a set of state-level demographic characteristics. This result is consistent across alternative specifications that model temporal and spatial correlations and nonlinear associations (eTable 2 in Supplement 1). We also performed supplemental analyses of the sensitivity of these results to measurement error and bias in maltreatment fatality measurement and found no support for a negative association between foster care and maltreatment mortality rates under any of our simulated measurement error scenarios (eFigures 1 and 2 in Supplement 1).

**Table.** Two-Way Fixed-Effects Regression of US Maltreatment Death Rate and Foster Care Entry Rate^a^

Variable	β (SE) [95% CI]
Foster care entry	0.17 (0.08) [0.01 to 0.34]
Less than ninth grade education	–0.12 (0.37) [–0.87 to 0.63]
Unemployed	–0.10 (0.15) [–0.41 to 0.20]
In poverty	0.02 (0.15) [–0.28 to 0.32]

^a^ Missing outcome values (n = 6) are imputed from 20 imputations.

## Discussion

Despite public concerns that decreasing foster care entries have led to increases in child fatalities, we found no evidence to support claims of a negative association between rates of foster care entry and rates of child maltreatment mortality rates at the state-year level. Importantly, our population-level analysis does not examine whether foster care placement for an individual child would reduce their fatality risk. Our results, however, suggest that marginal changes in foster care entry rates are not negatively associated with rates of child maltreatment mortality. Stated more directly, we found no evidence that an increase in a state’s rate of foster care entry is associated with a decrease in its rate of child maltreatment mortality and no evidence that a decrease in a state’s rate of foster care entry is associated with an increase in its rate of child maltreatment mortality.

These findings add to the increasing evidence of how foster care is associated with child, family, and population health. Research in this area is critical given recent policy reforms that prioritize preventing foster care entries, most notably the Family First Prevention Services Act.^11^ Maltreatment fatalities are one outcome among many potentially related to foster care entry dynamics, and current research suggests that decreasing foster care use may have both beneficial and adverse effects for child and family well-being.^24,25,26,27,28^ We encourage further research in this area and note that our findings do not rule out that there may be subpopulations of children for whom there is a negative association between foster care entry and fatality risk.^10^

In the urgent effort to reduce child maltreatment fatalities, our results encourage caution around advocating for states to remove more children from their homes into foster care. Highly publicized child fatalities typically lead to child protection responses that lower the threshold for foster care, leading state agencies to remove more children.^5,6,29,30^ Our findings suggest that this response may not be an effective fatality prevention strategy. Other research has identified broad policies that target public health and family well-being as offering promising alternatives. Analyzing the association between state spending on public benefit programs and child maltreatment fatalities, Puls and colleagues^31^ found that for each additional $1000 spent per person in poverty, expected mortality rates decrease by approximately 8%. Given clear causal links between income supports and child and family well-being,^32,33,34,35,36,37^ antipoverty programs may be an effective tool to reduce mortality rates. Recommendations from the World Health Organization, the American Academy of Pediatrics, and the American Public Health Association^38,39,40^ point to public health approaches, coupled with supports from medical and social service professionals for families at risk,^34,41,42,43^ as promising means of reducing fatal child maltreatment.

### Limitations

This study has several limitations. First, data on child maltreatment fatalities are imperfect. NCANDS data on such fatalities are likely underreported.^3,4,18,44^ NCANDS data on child maltreatment fatalities are submitted by individual states’ child protection agencies, which vary in their standards for identification and reporting. Although states are expected to consult other sources (such as vital statistics departments and medical examiners’ offices) to ensure their data are as complete as possible, processes for compiling these data vary.^1,17,45^ Although child fatality review programs are present in all 50 US states, the inclusion of review findings in NCANDS fatality counts varies across states.^44,45^ Moreover, identifying fatalities attributed to maltreatment as opposed to other causes often involves incomplete information and discretionary decision-making. We estimate our focal models using a subset of states that have more expansive reporting practices for fatalities (eTable 4 in Supplement 1) and build simulations to attempt to adjust for these likely features of the data (eTable 5 and eFigure 1 in Supplement 1). We discuss these measurement concerns and alternative data sources in more detail in eAppendix 4 in Supplement 1.

Second, although we did not find an association between the rates of entry into foster care and child maltreatment mortality rates at the population level, we are unable to examine causal relationships. The chief threats to causal identification in our analysis stem from omitted or unobservable processes varying within states over time or across states within years in ways that are correlated with foster care entry rates. An example of the former might be changes in the needs of children in the foster care system relative to the resources and capabilities of state systems, which could theoretically impact maltreatment mortality. An example of the latter is differential timing or duration of state responses to the COVID-19 pandemic, which could have impacted both foster care entries and maltreatment mortality. Future research might leverage exogenous policy or practice changes to analyze whether changes in foster care subsequently shift child maltreatment mortality rates.

## Conclusions

In this cross-sectional study, child maltreatment mortality rates were not negatively associated with foster care entry rates at the state level between 2010 and 2023. These descriptive results suggest that current evidence does not support the claim that reducing the number of children placed into foster care has increased child maltreatment mortality rates.

## Notes

Supplement 1. **eAppendix 1.** Sources of data on child maltreatment fatalities **eAppendix 2.** Alternative sources of data on child maltreatment fatalities **eAppendix 3**. Sensitivity of regression results to model specification **eAppendix 4.** Impacts of simulated bias and measurement error on regression results **eTable 1.** Data sources for counts of child maltreatment fatalities used in main analyses **eTable 2.** Sensitivity regressions of maltreatment death rate (per 100,000) on foster care entry rate (per 1,000), U.S. States 2010-2023 **eTable 3.** Sensitivity of main results to fixed effects, linear regressions of maltreatment death rate (per 100,000) on foster care entry rate (per 1,000), U.S. States 2010-2023 **eTable 4.** Sensitivity regression of maltreatment death rate (per 100,000) on foster care entry rate (per 1,000) tincluding a data quality indicator, U.S. States 2010-2023 **eTable 5.** Defining potential configurations of measurement error and bias in mortality measurement **eFigure 1.** Densities of simulated versus observed data under varying configurations of bias. Densities of observed and simulated data, with mean as dashed line **eFigure 2.** Regression parameter for the relationship between foster care entry (per 1,000 children) and child maltreatment mortality (per 100,000 children) rates under varying configurations of measurement error and bias in maltreatment mortality
eReferences

Supplement 2.
Data Sharing Statement
