# Supplementary material for: Foster Care and Child Maltreatment Mortality Rates in the US
Source: JAMA Netw Open. 2025 Dec 30;8(12):e2551677. doi: 10.1001/jamanetworkopen.2025.51677 (PMC12754682; doi:10.1001/jamanetworkopen.2025.51677)
Supplement: Supplement 1. — eAppendix 1. Sources of data on child maltreatment fatalities eAppendix 2. Alternative sources of data on child maltreatment fatalities eAppendix 3. Sensitivity of regression results to model specification eAppendix 4. Impacts of simulated bias and measurement error on regression results eTable 1. Data sources for counts of child maltreatment fatalities used in main analyses eTable 2. Sensitivity regressions of maltreatment death rate (per 100,000) on foster care entry rate (per 1,000), U.S. States 2010-2023 eTable 3. Sensitivity of main results to fixed effects, linear regressions of maltreatment death rate (per 100,000) on foster care entry rate (per 1,000), U.S. States 2010-2023 eTable 4. Sensitivity regression of maltreatment death rate (per 100,000) on foster care entry rate (per 1,000) tincluding a data quality indicator, U.S. States 2010-2023 eTable 5. Defining potential configurations of measurement error and bias in mortality measurement eFigure 1. Densities of simulated versus observed data under varying configurations of bias. Densities of observed and simulated data, with mean as dashed line eFigure 2. Regression parameter for the relationship between foster care entry (per 1,000 children) and child maltreatment mortality (per 100,000 children) rates under varying configurations of measurement error and bias in maltreatment mortality eReferences [file jamanetwopen-e2551677-s001.pdf]

Supplemental Online Content

Edwards F, Fong K, Apel R. Foster care and child maltreatment mortality rates in the US. *JAMA Netw Open*. 2025;8(12):e2551677.
doi:10.1001/jamanetworkopen.2025.51677

eAppendix 1. Sources of data on child maltreatment fatalities

eAppendix 2. Alternative sources of data on child maltreatment fatalities

eAppendix 3. Sensitivity of regression results to model specification

eAppendix 4. Impacts of simulated bias and measurement error on regression results

eTable 1. Data sources for counts of child maltreatment fatalities used in main analyses

eTable 2. Sensitivity regressions of maltreatment death rate (per 100,000) on foster care entry rate (per 1,000), U.S. States 2010-2023

eTable 3. Sensitivity of main results to fixed effects, linear regressions of maltreatment death rate (per 100,000) on foster care entry rate (per 1,000), U.S. States 2010-2023

eTable 4. Sensitivity regression of maltreatment death rate (per 100,000) on foster care entry rate (per 1,000) tincluding a data quality indicator, U.S. States 2010-2023

eTable 5. Defining potential configurations of measurement error and bias in mortality measurement

eFigure 1. Densities of simulated versus observed data under varying configurations of bias. Densities of observed and simulated data, with mean as dashed line

eFigure 2. Regression parameter for the relationship between foster care entry (per 1,000 children) and child maltreatment mortality (per 100,000 children) rates under varying configurations of measurement error and bias in maltreatment mortality

eReferences

This supplemental material has been provided by the authors to give readers additional information about their work.

© 2025 Edwards F et al. *JAMA Network Open.*

eAppendix 1. Sources of data on child maltreatment fatalities

States occasionally revise their submitted data on fatalities as investigations and reviews are completed. As Cohen notes, these revisions often reflect completed investigations and new information and routinely increase the total number of fatalities identified within a state for a given year[1]. To ensure that we use the most inclusive counts possible, we include only those fatality counts that reflect the most recent revision submitted to the U.S. Children's Bureau by the states. To do so, we evaluate the version number and release notes for all years of the NCANDS Agency File and retain counts for analysis when the release version is greater than one. For all years between 2014 and 2023, NCANDS Agency File data have been revised at least once; we use these updated data for all analyses.

eTable 1. Data sources for counts of child maltreatment fatalities used in main analyses

Data year	NCANDS Agency File version	Source utilized
2010	1	Child Maltreatment 2014
2011	1	Child Maltreatment 2015
2012	1	Child Maltreatment 2016
2013	1	Child Maltreatment 2017
2014	3	NCANDS Agency file 2014
2015	4	NCANDS Agency file 2015
2016	4	NCANDS Agency file 2016
2017	5	NCANDS Agency file 2017
2018	4	NCANDS Agency file 2018
2019	4	NCANDS Agency file 2019
2020	5	NCANDS Agency file 2020
2021	4	NCANDS Agency file 2021
2022	3	NCANDS Agency file 2022
2023	2	NCANDS Agency file 2023

For cases where the data have not been revised (version = 1) in years 2010 through 2013, we instead rely on the most recently published fatality counts published by the U.S. Children's Bureau in the annual 'Child Maltreatment' report, Table 4-2[2]. These reports provide fatality counts for the year of release but also provide a table of fatality counts for the five prior reporting years, reflecting any revisions that states made to their fatality counts. We exclusively use the trailing year in these five-year windows; for 2010 data we look to the 2014 'Child Maltreatment report', for 2011 we use the 2015 report and so on. For years where NCANDS Agency File counts have not been revised, we use these counts transcribed from Table 4-2 in the 'Child Maltreatment' reports. We use the 'Child Maltreatment' report fatality counts for years 2010 – 2013, and NCANDS Agency File fatality counts for 2014 through 2023. We show the data version of the NCANDS Agency File and our selected source of data in eTable 1.

eAppendix 2. Alternative sources of data on child maltreatment fatalities

Many have noted that current child maltreatment fatality data likely undercount the true number of fatalities involving child abuse or neglect[1,3–5]. We agree that measurement error and bias are a significant concern for any analysis of statistical patterns in maltreatment related deaths. In this analysis, we rely on the most recent revisions of data provided by states to the U.S. federal government (see eTable 1), but these counts still likely underestimate the incidence of maltreatment fatalities.

There is not currently an alternative to NCANDS for a nationally representative source of data on child maltreatment fatalities that can be used for population-level inference. Some scholars have turned to the National Violent Death Reporting System (NVDRS) to capture maltreatment-related fatalities[6]. While these data offer a high-level of detail for included cases, they are restricted to deaths identified as homicides. Neglect is often a primary or major contributor to maltreatment fatalities[2,7]. Maltreatment death counts derived from NVDRS are dramatically lower than those provided in NCANDS, with a recent study estimating 285 deaths over a ten-year period. NCANDS identifies about 20,000 deaths over a similar period. While the National Vital Statistics System may offer a promising alternative, and one we considered at length for this analysis, there is no set of ICD-10 codes that are

© 2025 Edwards F et al. *JAMA Network Open.*

adequately sensitive to detect the broad array of proximate causes that may be identified as child maltreatment fatalities[8].

Child and infant death review programs provide in-depth assessments of the circumstances and contexts of deaths of children, often expansively incorporating information that may not be documented in records compiled by a state child welfare agency[3,9–11]. These death review teams often identify maltreatment related causes of death that had not been previously identified by child welfare systems. Child death reviews are completed in all fifty states. The National Fatality Review Case Reporting System (NFR-CRS) collects data from child and infant fatality review teams and makes de-identified data available to the research community. The National Center for Fatality Review and Prevention (CFRP) removes personally identifying information from data prior to release, including the state in which a death occurred. This de-identification procedure forecloses state-level analysis of the sort we conduct in this study. Additionally, CFRP advises that "[t]he most significant limitation is that most states do not review and enter all fetal, infant, or child deaths. As a result, the NFR-CRS data are not population-based and cannot be used to calculate rates. Additionally, the NFR-CRS data cannot be compared between states or local teams or over time because of the variation in deaths reviewed, the timing of reviews, and fluctuating participation by states"[10].

Individual states occasionally publish summary statistics on outcomes of child fatality reviews. For example, Michigan has published counts of child abuse and neglect deaths derived from child fatality review processes. In Chart 44 of the report 'Child Deaths in Michigan', authors identify 48 maltreatment deaths in 2015, 45 in 2016, 38 in 2017, 33 in 2018, and 49 in 2019[12]. For comparison, the NCANDS Agency File data we use in focal analyses reports that Michigan had 83 maltreatment deaths in 2015, 86 in 2016, 51 in 2017, 49 in 2018, and 63 in 2019. NCANDS data captured more deaths than those reported by Michigan's Child Death Review team for each of these years.

The Child and Family Services Improvement and Innovation Act (P.L. 112–34) of 2011 required states to provide more detailed information on child fatalities to the federal government. Many states that had not previously included data from child fatality review teams, medical examiners, and other sources in their NCANDS fatality counts began to do so. In a review of state addenda to the 'Child Maltreatment 2022' report that detail state procedures for identifying and reporting maltreatment fatalities, we identified 30 states that describe the inclusion of data external to the state's child welfare agency in fatality counts. Most of these 30 cases explicitly identify child fatality review teams as a source of maltreatment fatality data reported to NCANDS. We develop a sensitivity analysis of our main results to state data quality inclusion criteria displayed in eTable 4; results are substantively unchanged when compared to our focal estimates shown in Table 1.

A team of child welfare scholars have recently begun an independent data collection effort to improve measurement of child maltreatment fatalities[13]. While this effort has promise to improve measurement in years to come, at present it does not extend the scope of national data availability on maltreatment fatalities. The bulk of fatalities reported in the team's CANDID database appear to overlap substantially with the cases identified in NCANDS, and the magnitude of reported fatality frequencies are similar to those derived from NCANDS.

To summarize, despite the limitations of the maltreatment fatality data collected by the U.S. Children's Bureau, NCANDS remains the most comprehensive source of information on abuse and neglect deaths, and the only source with national coverage of the theoretical population of all children in the United States. We consider the sensitivity of our conclusions to possible measurement error and bias in NCANDS, as well as the sensitivity of our results to variation in state reporting standards in supplemental analyses below (see eFigure 1, eFigure 2, and eTable 5).

eAppendix 3. Sensitivity of regression results to model specification

We expand our focal two-way fixed effects model several ways to probe for sensitivity. First, we allow for serial dependence that is not accounted for by the state fixed effects. This method uses Prais-Winsten regression to estimate a first-order AR(1) correlation term. Second, we allow for geographic dependence using a spatial autoregressive model. This method obtains a spatial weight matrix with first-order contiguity, SAR(1), meaning neighboring states are assumed to share unobserved commonality. We separately allow second-order contiguity to account for broader regional similarities, but do not report these results since they are similar to SAR(1). Third, we estimate an exponential-mean model using Poisson pseudo-maximum likelihood, including state and year fixed effects.

Results are displayed in eTable 2. In our focal two-way fixed effects regression with cluster robust standard errors (Model 1), we find a point estimate for the relationship between foster care entry and maltreatment fatalities of 0.17 (CI [0.01, 0.34]). In Model 2, a Prais-Winsten regression that includes an autoregressive term to adjust for serial correlation, we estimate the association at 0.17 [0.02, 0.32]. In Model 3, which includes a spatial error term for

© 2025 Edwards F et al. *JAMA Network Open.*

dependencies between neighboring states, we again find a small positive association [0.018, 0.24] between foster care entry and maltreatment mortality. In Model 4, a Poisson regression employing a log link function, we estimate the parameter describing the relationship between foster care entry and maltreatment fatalities at 0.26 [-0.09, 0.61]. We find no evidence that the focal relationship between foster care entry rates and maltreatment mortality rates is sensitive to these alternative model specifications in a manner that has substantive implications for our conclusions.

eTable 2. Sensitivity regressions of maltreatment death rate (per 100,000) on foster care entry rate (per 1,000), U.S. States 2010-2023

	(1)<br>Coef. (Std. Err.)	(2)<br>Coef. (Std. Err.)	(3)<br>Coef. (Std. Err.)	(4)<br>Coef. (Std. Err.)
Foster Care Entry	+0.17 (0.08) *	+0.17 (0.08) *	+0.17 (0.06) **	+0.26 (0.18)
% < 9th Grade	–0.12 (0.37)	–0.02 (0.21)	–0.10 (0.14)	–0.63 (0.60)
% Unemployed	–0.10 (0.15)	–0.11 (0.12)	–0.09 (0.08)	–0.46 (0.48)
% in Poverty	+0.02 (0.15)	+0.01 (0.12)	+0.02 (0.09)	+0.82 (0.96)
Unit Fixed Effects?	Y	Y	Y	Y
Time Fixed Effects?	Y	Y	Y	Y
Serial Correlation?	N	Y	N	N
Spatial Correlation?	N	N	Y	N

Note: NT = 700. All models include fixed effects for states and years, along with controls for the percentage of adults 25+ with less than 9th grade education, unemployment rate, and poverty rate. Missing outcome values are imputed from 20 imputations. Model 1 is the standard TWFE estimator. Model 2 includes an AR(1) error term from Prais-Winsten regression. Model 3 includes a spatial error from a first-order contiguity matrix. Model 4 is Poisson pseudo-maximum likelihood. In this model, the foster care entry rate is logged, so the coefficient is an elasticity. Standard errors are clustered by state and year except for Model 3.
** p < 0.05; ** p < 0.01; *** p < 0.001 (two-sided tests).*

We also evaluate the sensitivity of our results to model specification by estimating a nested set of regressions that evaluate the impact of both fixed effects terms and demographic controls on our conclusions; results are displayed in eTable 3. We estimate separately an unconditional regression of foster care entries per 1,000 population on maltreatment mortality rates per 100,000 in model (1) and find no association between the two measures. In model (2) we include fixed effects for states, which adjust for time stable features of each state correlated with foster care and maltreatment mortality, again finding no association. In model (3) we adjust for year fixed effects, which control for time-varying national processes related to both foster care and maltreatment and find no association. In model (4) we include both state and year fixed effects, but omit demographic predictors, we find a point estimate for the relationship similar to that we observe in our fully elaborated model (see Table 1), albeit with a larger standard error. Conditioning on unobserved state and year characteristics does not substantively change our estimate of the relationship between foster care entry rates and child maltreatment mortality rates.

eTable 3. Sensitivity of main results to fixed effects, linear regressions of maltreatment death rate (per 100,000) on foster care entry rate (per 1,000), U.S. States 2010-2023

	(1)<br>Coef. (Std. Err.)	(2)<br>Coef. (Std. Err.)	(3)<br>Coef. (Std. Err.)	(4)<br>Coef. (Std. Err.)
Foster Care Entry	-0.01 (0.07)	+0.01 (0.06)	+0.01 (0.07)	+ 0.18 (0.09)
Unit FE?	N	Y	N	Y
Time FE?	N	N	Y	Y

Note: NT = 700. Standard errors are unclustered in (1), clustered by state in (2), clustered by year in (3), and clustered by state and year in (4).
* p < 0.05; ** p < 0.01; *** p < 0.001 (two-sided tests).

We also evaluate the sensitivity of our results to variation in data collection and reporting practices across states**.** States provide details about their inclusion criteria and reporting practices for maltreatment fatalities in addenda to the Children's Bureau's annual 'Child Maltreatment' reports. We use this information, coupled with information from the NCANDS Agency File to develop a binary measure indicating whether a state reports

© 2025 Edwards F et al. *JAMA Network Open.*

exclusively maltreatment fatalities involving a child protection investigation or utilizes a more expansive set of inclusion criteria. These expansive criteria generally include data from child death review teams, medical examiners, law enforcement, and other professional sources of information on child fatalities. We include this predictor in our focal two-way fixed effects regression; results are reported in eTable 4. Our estimate of the relationship between foster care entry rates and maltreatment mortality rates is similar in sign, magnitude, and uncertainty when compared to our focal estimates displayed in Table 1 in the manuscript or eTable 2 in this supplement. This evidence suggests that variation in data quality across states and within-states over time does not impact our substantive conclusions.

eTable 4. Sensitivity regression of maltreatment death rate (per 100,000) on foster care entry rate (per 1,000) while including a data quality indicator, U.S. States 2010-2023

	**Coef. (Std. Err.)**
Foster Care Entry	+0.17 (0.08)*
% < 9th Grade	-0.13 (0.37)
% Unemployed	-0.11 (0.15)
% in Poverty	+0.02 (0.15)
Inclusive mortality data practices (binary)	+0.13 (0.11)
Unit Fixed Effects?	Y
Time Fixed Effects?	Y

Note: NT = 700. All models include fixed effects for states and years, along with controls for the percentage of adults 25+ with less than 9th grade education, unemployment rate, and poverty rate. Missing outcome values are imputed from 20 imputations. Standard errors are clustered by state and year.
* p < 0.05; ** p < 0.01; *** p < 0.001 (two-sided tests).

eAppendix 4. Impacts of simulated bias and measurement error on regression results

Following Blackwell, Honaker, and King[14,15], we evaluate the potential impacts of measurement error on focal estimands through overimputation of observed data, a Monte Carlo simulation method[16]. We assume that there is some 'true' and unobserved child maltreatment mortality rate we call y^* that is a function of observed mortality rates y. Because mortality rates have a skewed non-negative distribution with some observations reported as a rate of 0, we take the natural logarithm of the observed data plus one prior to all transformations. We assume that bias in the observed data follows a simple additive functional form, where γ is a non-negative bias term. Because of the logarithmic transformation, this additive relationship becomes multiplicative and scaling relative to crude mortality rates.

$$E[ln(1 + y_i^*)] = ln(1 + y_i + \gamma)$$

We also assume that there is additional white noise (symmetric) stochastic error in measurement, parameterizing the standard deviation to create a margin of error approximately equal to 10 percent of the expected value of our bias adjusted mortality rate.

$$ln(1 + y^*) \sim Normal(ln(1 + y + \gamma), \sigma_{y^*}^2)$$

$$\sigma_{y_i^*} = 0.05 \times ln(1 + y_i + \gamma)$$

Our bias term takes the form γ. We develop five scenarios for bias in mortality estimates, reflecting increasing degrees of negative bias in observed data. We display the impact of our varying assumptions about bias on maltreatment mortality on the median of the empirical data in eTable 5 and eFigure 1. At the median, the minimal bias scenario inflates the observed value by about 5 percent; the low bias scenario inflates the median by about 13 percent; the medium bias scenario inflates the median by about 25 percent; the high bias scenario inflates

© 2025 Edwards F et al. *JAMA Network Open.*

the median by about 51 percent, and the extreme bias scenario inflates the median by about 76 percent. This range of hypothetical bias is in line with prior estimates of the magnitude of potential bias in NCANDS fatality counts[1].

eTable 5. Defining potential configurations of measurement error and bias in mortality measurement

Scenario	γ	Impact of transformation on observed median maltreatment mortality rate
No bias (Observed data)	0	1.97
Minimal bias	0.1	2.07
Low bias	0.25	2.22
Medium bias	0.5	2.47
High bias	1.0	2.97
Extreme bias	1.5	3.47

The distributions of these simulated values of y^* are displayed relative to the distribution of the observed data y in eFigure 1. We estimate 10 overimputations of our data over each of the 20 datasets used in the main analysis (missing data imputation addresses n=6 missing cases with 20 imputed datasets). This results in 20 'observed' versions of the data, and 200 simulated datasets for each scenario. Note that the mean of the distribution shifts rightward as the magnitude of induced bias increases, as expected. Note also a large shift in the proportion of cases with mortality rates greater than or equal to 2 deaths per 100,000 children as the magnitude of bias increases. The dashed line indicates the change in the mean mortality rate under each simulated bias scenario.

We use each of these 1200 imputed datasets to fit our focal two-way fixed effects regression. Here, we estimate a similar model, but substitute y^*_{jt} on the left-hand side of the equation. This variable represents overimputed state-year mortality rates under each scenario of potential measurement error and bias. This model allows us to evaluate whether and how the focal relationship between foster care entry and maltreatment-attributed mortality rates is impacted under varying configurations of error and bias.

$$y^*_{jt} = \alpha + \beta X_{it} + \theta Z_{it} + u_j + u_t + e_{jt}$$

We estimate models across each imputed dataset using Hamiltonian Monte Carlo with regularizing priors, then pool posterior estimates of β across model fits and visualize their densities for each scenario in eFigure 2. These results suggest that the substantive conclusions we draw from our focal model would not change under any of our specified scenarios of bias and error. Under each condition, we find no clear support for a negative direction for β. As in our main analysis, 95 percent posterior densities of β under each scenario are generally centered at small positive values. This evidence suggests that even under conditions of severe bias and measurement error, we would not expect to estimate a negative relationship between foster care entry rates and child maltreatment mortality rates.

© 2025 Edwards F et al. *JAMA Network Open.*

eFigure 1. Densities of simulated versus observed data under varying configurations of bias. Densities of observed and simulated data, with mean as dashed line

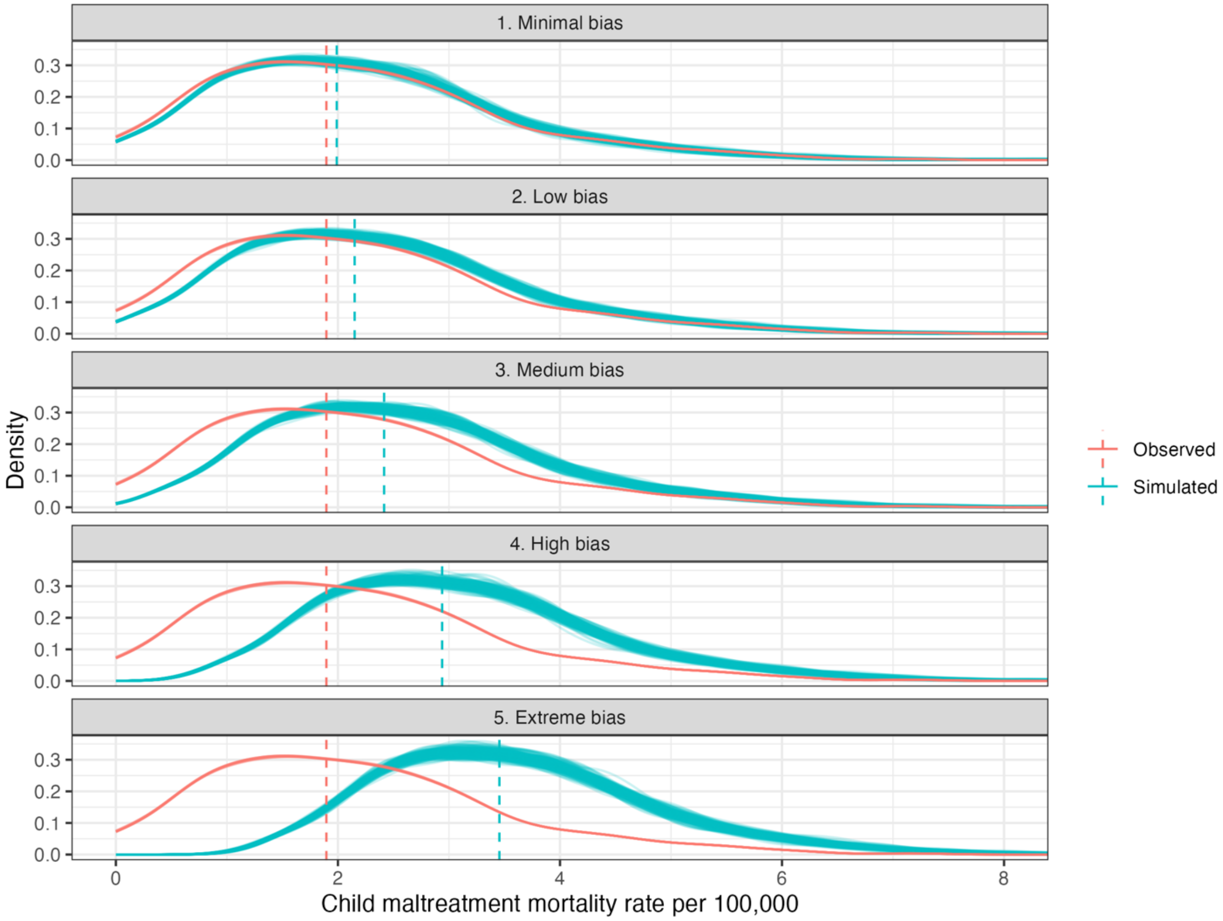

© 2025 Edwards F et al. *JAMA Network Open.*

eFigure 2. Regression parameter for the relationship between foster care entry (per 1,000 children) and child maltreatment mortality (per 100,000 children) rates under varying configurations of measurement error and bias in maltreatment mortality

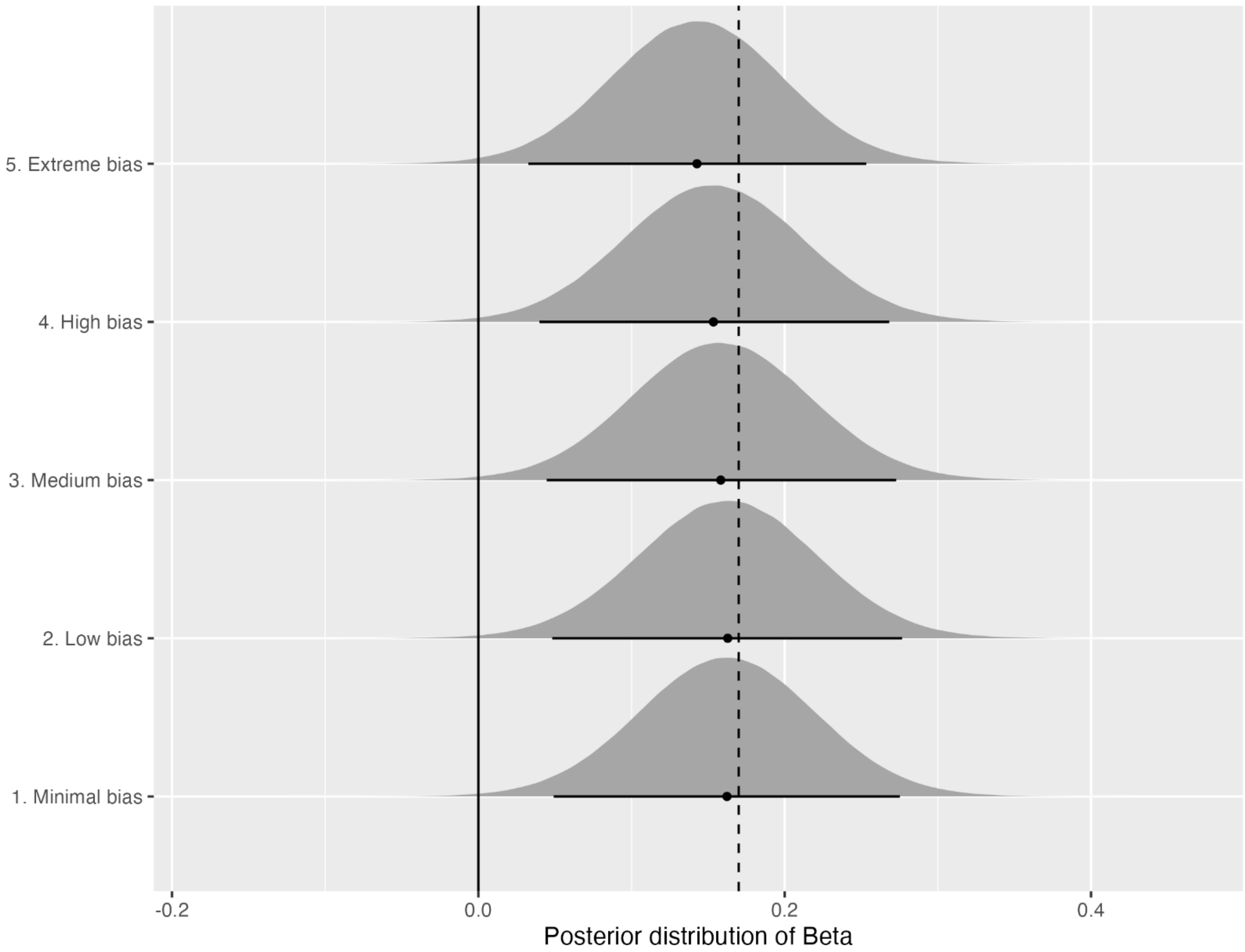

Posterior distributions of two-way fixed effect Beta parameter on simulated maltreatment mortality rate data under varying measurement error configurations

© 2025 Edwards F et al. *JAMA Network Open.*

eReferences

1. Cohen M. *A Jumble of Standards: How State and Federal Authorities Have Underestimated Child Maltreatment Fatalities*. American Enterprise Institute; 2024. Accessed October 30, 2025. https://www.aei.org/wp-content/uploads/2024/05/A-Jumble-of-Standards.pdf?x85095
2. US Children's Bureau. *Child Maltreatment*. US Department of Health and Human Services; 2024. Accessed September 20, 2024. https://www.acf.hhs.gov/cb/data-research/child-maltreatment
3. Warren MD, Pilkey D, Joshi DS, Collier A. Fetal, Infant, and Child Death Review: A Public Health Approach to Reducing Mortality and Morbidity. *Pediatrics*. 2024;154(Supplement 3):e2024067043B. doi:10.1542/peds.2024-067043B
4. Commission to Eliminate Child Abuse and Neglect Fatalities. *Within Our Reach: A National Strategy to Eliminate Child Abuse and Neglect Fatalities*. Government Printing Office; 2016. Accessed September 20, 2024. https://www.acf.hhs.gov/cb/report/within-our-reach-national-strategy-eliminate-child-abuse-and-neglect-fatalities
5. Putnam-Hornstein E, Wood JN, Fluke J, Yoshioka-Maxwell A, Berger RP. Preventing severe and fatal child maltreatment: making the case for the expanded use and integration of data. *Child Welfare*. 2013;92(2):59-75.
6. Michaels NL, Letson MM. Child maltreatment fatalities among children and adolescents 5–17 years old. *Child Abuse & Neglect*. 2021;117:105032. doi:10.1016/j.chiabu.2021.105032
7. Palusci VJ, Bishop PL. What have we learned about child maltreatment fatality prevention? *APSAC Advisor*. 2025;38(1).
8. Garza HH, Piper KE, Barczyk AN, Pérez A, Lawson KA. Accuracy of ICD-10-CM coding for physical child abuse in a paediatric level I trauma centre. *Injury Prevention*. 2021;27(Suppl 1):i71-i74. doi:10.1136/injuryprev-2019-043513
9. Palusci VJ. Comparing types of child fatality review in the U.S. *Child Protection and Practice*. 2024;2:100040. doi:10.1016/j.chipro.2024.100040
10. Collier A, Dykstra H, Shaw E, Fournier R, Schnitzer P. National Fatality Review Case Reporting System: Twenty Years of Data Collection. *Pediatrics*. 2024;154(Supplement 3):e2024067043C. doi:10.1542/peds.2024-067043C
11. McCarroll JE, Fisher JE, Cozza SJ, Whalen RJ. Child Maltreatment Fatality Review: Purposes, Processes, Outcomes, and Challenges. *Trauma, Violence, & Abuse*. 2021;22(5):1032-1041. doi:10.1177/1524838019900559
12. Hertel E. *Child Deaths in Michigan: A Report on Case Reviews Conducted from 2015 to 2019*. Michigan Public Health Institite; 2022. https://www.mifrp.org/wp-content/uploads/Michigan-Child-Death-State-Advisory-Team-Report-on-Reviews-Conducted-in-2015-2019.pdf
13. Schaefer Riley N, Putnam-Hornstein E, Font S. About Us - Lives Cut Short. Lives Cut Short. Accessed October 31, 2025. https://livescutshort.org/about-us/
14. Blackwell M, Honaker J, King G. A Unified Approach to Measurement Error and Missing Data: Details and Extensions. *Sociological Methods & Research*. 2017;46(3):342-369. doi:10.1177/0049124115589052
15. Blackwell M, Honaker J, King G. A Unified Approach to Measurement Error and Missing Data: Overview and Applications. *Sociological Methods & Research*. 2017;46(3):303-341. doi:10.1177/0049124115585360
16. Banack HR, Hayes-Larson E, Mayeda ER. Monte Carlo Simulation Approaches for Quantitative Bias Analysis: A Tutorial. *Epidemiologic Reviews*. 2021;43(1):106-117. doi:10.1093/epirev/mxab012

© 2025 Edwards F et al. *JAMA Network Open.*
