# Supplementary material for: Foster Care and Child Maltreatment Mortality Rates in the US
Source: JAMA Netw Open. 2025 Dec 30;8(12):e2551677. doi: 10.1001/jamanetworkopen.2025.51677 (PMC12754682; doi:10.1001/jamanetworkopen.2025.51677)
Supplement: Supplement 2. — Data Sharing Statement [file jamanetwopen-e2551677-s002.pdf]

Data Sharing Statement

Edwards. Foster Care and Child Maltreatment Mortality Rates in the US. *JAMA Netw Open*. Published December 30, 2025. doi:10.1001/jamanetworkopen.2025.51677

Data
Data available: Yes
Data types: Data (not involving human participants)
How to access data: Secondary data collected by federal government. Data available by request from the National Data Archive on Child Abuse and Neglect. Data is available by request to the research community with a signed data access agreement.
When available: With publication

Supporting Documents
Document types: Statistical/analytic code
How to access documents: All code and data access details provided at https://osf.io/pg6m2
When available: With publication

Additional Information
Who can access the data: Public
Types of analyses: All analyses will be made available
Mechanisms of data availability: Data is available by request to the research community with a signed data access agreement.
